# Clinical impact of prevalence and severity of COPD on the decision-making process for therapeutic management of lung cancer patients

**DOI:** 10.1186/1471-2466-14-14

**Published:** 2014-02-05

**Authors:** Naozumi Hashimoto, Asuka Matsuzaki, Yu Okada, Naoyuki Imai, Shingo Iwano, Kenji Wakai, Kazuyoshi Imaizumi, Kohei Yokoi, Yoshinori Hasegawa

**Affiliations:** 1 Department of Respiratory Medicine, Nagoya University Graduate School of Medicine, 65 Tsurumai-cho, Showa-ku, Nagoya 466-8550, Japan; 2 Department of Radiology, Nagoya University Graduate School of Medicine, Nagoya, Japan; 3 Department of Preventive Medicine, Nagoya University Graduate School of Medicine, Nagoya, Japan; 4 Department of Respiratory Medicine and allergy, Fujita Health University, Toyoake, Japan; 5 Department of Thoracic Surgery, Nagoya University Graduate School of Medicine, Nagoya, Japan

**Keywords:** Chronic obstructive lung disease, Bronchoscopy, Spirometry screening assessment, Thoracic surgery, Japanese population

## Abstract

**Background:**

Recent studies suggest that coexistence of chronic obstructive pulmonary disease (COPD) might be independently related to a worse prognosis for lung cancer. However, because data on the substantial prevalence of COPD and its severity in Asian lung cancer patients remain limited, clinical impact of prevalence and severity of COPD among the population has not been fully evaluated. Furthermore, patients with COPD often have comorbidities. Thus, whether the decision-making process for therapeutic management of lung cancer patients might be independently affected by COPD remains elusive.

**Methods:**

Clinical impact of prevalence and severity of COPD were evaluated in 270 Japanese patients with newly diagnosed lung cancer who were sequentially registered and underwent bronchoscopy from August 2010 to July 2012 at Nagoya University hospital. Furthermore, to explore whether or not the severity of airflow obstruction might affect the decision to propose thoracic surgery with curative intent, we evaluated data from patients with lung cancer at stage 1A to 3A who underwent spirometry and bronchoscopy.

**Results:**

The prevalence rate of COPD was 54.4% among Japanese patients with lung cancer who underwent bronchoscopy. The incidence of Global Initiative for Chronic Obstructive Lung Disease (GOLD) grades 1 and 2 was significantly higher than that of GOLD grade 3. Although COPD-related comorbidities were not independent factors for proposing thoracic surgery, the number of thoracic surgeries performed was significantly less in the COPD group than the non-COPD group. Multivariate analysis showed that more severe airway obstruction, advanced clinical staging, and higher age, were independent factors associated with the decision on thoracic surgery.

**Conclusions:**

We demonstrated a high prevalence of COPD among Japanese lung cancer patients. Based on the knowledge that severity of COPD is one of the most important factors in the therapeutic decision, comprehensive assessment of COPD at bronchoscopy might allow us to implement the optimum management for lung cancer patients.

## Background

Chronic obstructive pulmonary disease (COPD) and lung cancer are projected to continue to increase the burden of disease worldwide until 2020 [1,2]. Many studies suggest that COPD might be independently related to a worse prognosis for lung cancer [3-6]. A recent review suggests more inclusive consideration for surgical resection with curative intent in lung cancer patients with COPD because limited surgical resections or nonsurgical therapeutic options might provide inferior survival compared with resection [6]. However, because older patients with COPD are often known to have much lower pulmonary function as well as other comorbidities [7,8], whether or not the decision-making process for therapeutic management of lung cancer patients might be independently affected by the coexistence and severity of COPD remains elusive. Recent studies show that the prevalence of COPD is 40-70% of smokers diagnosed with lung cancer [9,10]. On the other hand, many studies suggest that the increasing incidence of adenocarcinoma in Asian populations including the Japanese population might be associated with epidermal growth factor receptor (EGFR) mutation rather than with smoking [11,12]. Zhang, et al. show a low prevalence of COPD in hospitalized lung cancer patients in China (21.6%; 705/3263 cases) [13], whereas we recently demonstrated that the prevalence of COPD was more than 40% in Japanese patients undergoing thoracic surgery [14]. Because another therapeutic option besides surgery, such as chemotherapy and/or radiation, might be selected for the older lung cancer patients with COPD [8], substantial prevalence of COPD among Asian patients with newly diagnosed lung cancer might be higher than that in our study [14]. Thus, data on the prevalence of COPD and its severity in Asian populations remain limited. Taken together, clinical impact of prevalence and severity of COPD among Asian lung cancer patients should be determined. Bronchoscopy is performed for most patients with lung cancer before the decision is made for treatment of lung cancer. In the present study, the association of COPD prevalence with lung cancer characteristics was evaluated by spirometry among 270 Japanese patients with lung cancer who underwent bronchoscopy. We also evaluated whether or not the prevalence and severity of COPD might independently affect the decision-making process for the treatment of lung cancer in this population.

## Methods

### Population

Patients with lung cancer who underwent bronchoscopy at Nagoya University hospital from August 2010 to July 2012 were the subjects of this retrospective study. The study was approved by the Institutional Review Board of Nagoya University Graduate School of Medicine (No 27-2) [14].

Information about patient characteristics, pathological diagnosis of lung cancers, and clinical staging of lung cancers, was obtained from hospital records as previously reported [14]. COPD-related systemic comorbidities were defined as diabetes, hypertension, hyperlipidemia, or ischemic cardiac disease [7]. Spirometry screening assessment was performed on admission of the patients undergoing bronchoscopy. Spirometry was performed with a calibrated dry spirometer, a FUDAC-77 (Fukuda Denshi Co., Ltd., Tokyo, Japan), according to the American Thoracic Society (ATS) standards applied in our hospital [15]. Subjects were assigned to the COPD group, if they had airflow obstruction as determined by an FEV1/FVC ratio was below 0.70, and the remaining subjects were assigned to the non-COPD group [9,10]. Reversibility, for which an improvement of 200 ml in FEV1 and 12% in FEV1 from baseline was defined as positive, was also evaluated 15 minutes after a short-acting beta2-agonist was given. Severity of airflow limitation in COPD was determined by using the Global Initiative for Chronic Obstructive Lung Disease (GOLD) grade [16]; that is, grade 1 (%FEV1 predicted >80%), grade 2 (50% <%FEV1 predicted <80%), grade 3 (30% <%FEV1 predicted <50%), and grade 4 (%FEV1 predicted <30%). When the patients did not undergo spirometry as a screening assessment at a bronchoscopy, data were collected from the preoperative pulmonary assessment for those undergoing thoracic surgery or from a spirometric assessment before the bronchoscopy. Information about the existence of emphysema from the chest computed tomography (CT) was obtained from radiological reports. Clinical staging of lung cancer was based on the tumor, node, metastasis (TNM) staging using the standards of the Union International Contre le Cancer (UICC), 7^th^ edition [17]. Histological type of adenocarcinoma, squamous cell carcinoma (Sq), large cell carcinoma (Large), small cell lung carcinoma (SCLC), and non-small cell lung carcinoma (NSCLC) was determined according to the World Health Organization’s classification [18]. We also collected information about the status of EGFR mutation in lung cancers from patient records.

### Statistical analysis

All data were checked for completeness, and the analyzed variables were tested for normality of distribution by the Shapiro-Wilk test. Normally distributed variables were compared by the t test and non-normally distributed ones by the Mann-Whitney test between the COPD and non-COPD group, or between the surgery and the non-surgery group. Comparisons between the percentages of patients with each GOLD grade or tumor type were made using the χ2 test or with Fisher’s exact test. Factors that were found to be predictive for relinquishing surgery in the above-mentioned univariate analyses were entered into a forward-and-backward stepwise logistic regression analysis to identify independent factors for the relinquishment of surgery. Statistical analyses were performed with PASW Statistics version 18.0 (SPSS Inc, Chicago, IL), and a P value of less than 0.05 was considered statistically significant.

## Results

### Demographic distribution of patient characteristics among the study population

**Figure 1:**
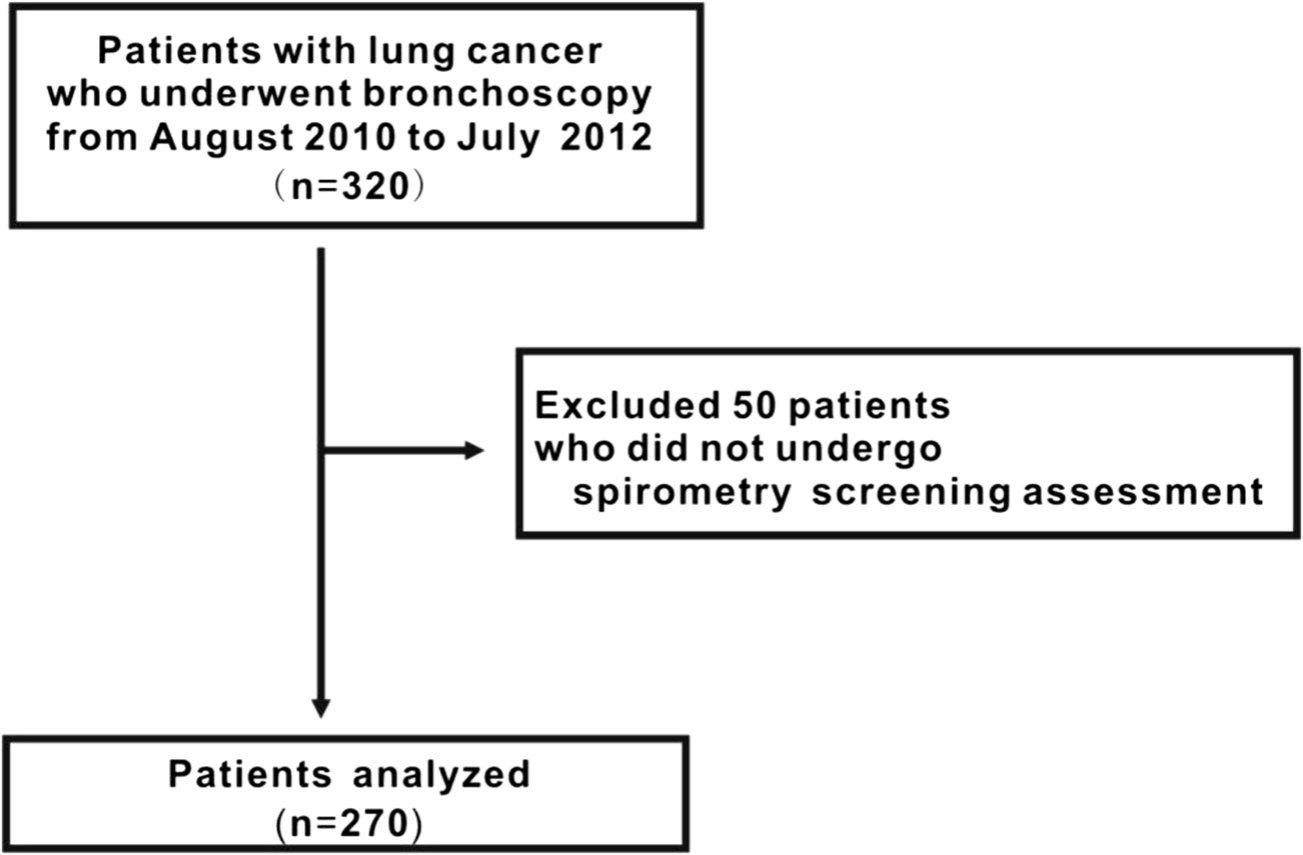
**Study profile.** Schematic diagram showing the study profile.

Data from 320 patients with lung cancer who were sequentially registered and underwent bronchoscopy from August 2010 to July 2012 were obtained from hospital records (Figure 1). Fifty patients who had not undergone pulmonary assessment by spirometry were subsequently excluded from the study population. In total, 234 patients with lung cancers underwent spirometry screening assessment at bronchoscopy. A further 36 patients with lung cancers did not undergo spirometry at bronchoscopy but received it at thoracic surgery or before bronchoscopy. Ultimately, the study population included 270 patients who underwent spirometry and bronchoscopy among 320 patients with lung cancer (84.4%) (Figure 1).

**Figure 2:**
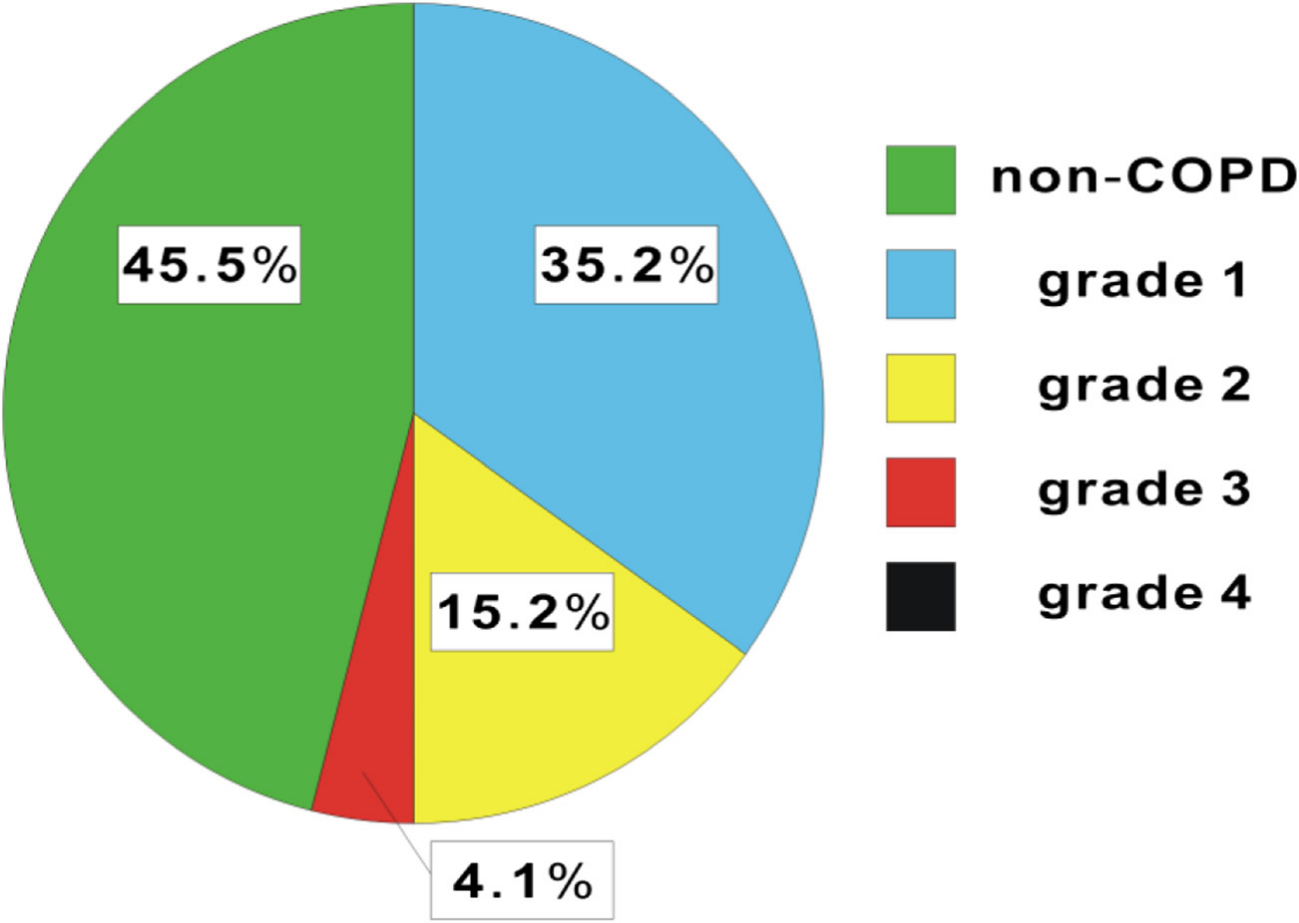
**Population of non-COPD and COPD in Japanese patients with lung cancer.** Schematic presentation of the percentage of non-COPD (n = 123) and COPD (n = 147) among Japanese patients with lung cancer. Patients with COPD were classified by GOLD grade, that is, grade 1 (n = 95), grade 2 (n = 41), grade 3 (n = 11), and grade 4 (n = 0).

In the present population, 54.4% of the patients who underwent bronchoscopy had COPD (147/270 cases). Table 1 shows a comparison of the patient characteristics among the study groups. Only 8.5% of the total study population had been managed as COPD. Overall, the 147 patients in the COPD group were older and more likely to be male, and to have a more extensive smoking history, as compared with the 123 patients in the non-COPD group. When we evaluated the severity of airflow obstruction in the COPD group, the incidence of GOLD grade 1 and 2 was significantly higher than that of GOLD grade 3 (Figure 2 and Table 2). We also evaluated chest CT findings to determine the involvement of emphysema. The percentage of the COPD group with involvement of emphysema in the chest CT findings was almost twice as high as that of the non-COPD group (38.8% vs 20.3%, respectively).

**Table 1:** Patient characteristics among non-COPD and COPD patients

	**All cases (n = 270)**	**Non-COPD (n = 123)**	**COPD (n = 147)**	**p value**
**Cases**	**100 (270)**	**45.6 (123)**	**54.4 (147)**	**0.0001**^**#**^
**Age, years**^**a**^	**70.1 (39–88)**	**67.9 (39–82)**	**71.9 (51–87)**	**0.0001**^**#**^
**Sex, male**	**73.7 (199)**	**62.6 (77)**	**83.0 (122)**	**0.0001**^**#**^
**History of smoking**	**78.9 (213)**	**65.9 (81)**	**89.8 (132)**	**0.0001**^**#**^
**COPD managed**^**b**^	**8.5 (23)**	**1.6 (2)**	**14.3 (21)**	**0.0001**^**#**^
**COPD-related systemic comorbidities**	**55.9 (151)**	**52.8 (65)**	**58.5 (86)**	**0.390**
**Diabetes**	**19.3 (52)**	**16.3 (20)**	**21.8 (32)**	**0.280**
**Ischemic cardiac disease**	**7.4 (20)**	**2.4 (3)**	**11.6 (17)**	**0.004**^**#**^
**Hypertension**	**38.1 (103)**	**37.4 (46)**	**38.8 (57)**	**0.900**
**Hyperlipidemia**	**11.9 (32)**	**8.1 (10)**	**15.0 (22)**	**0.092**

n indicates number.

^a^Data are shown as mean (range).

^b^indicates the patients who had been diagnosed as COPD before bronchoscopy.

All other data are shown as% (numbers).

^#^p < 0.05.

COPD: chronic obstructive pulmonary disease.

**Table 2:** Physical assessment variables among non-COPD and COPD patients

	**All cases (n = 270)**	**Non-COPD (n = 123)**	**COPD (n = 147)**	**p value**
**BMI (kg/m**^**2**^**)**^**a**^	**22.1 (2.8)**	**22.0 (2.8)**	**22.2 (2.9)**	**0.749**
**spirometric variables**				
**%VC**^**a**^	**105.9 (20.6)**	**104.8 (20.8)**	**106.7 (20.5)**	**0.520**
**FEV1 (ml)**^**a**^	**2062 (610)**	**2302 (555)**	**1861 (583)**	**0.0001**^**#**^
**FEV1/FVC**^**a**^	**67.3 (12.6)**	**77.8 (6.3)**	**58.9 (10.3)**	**0.0001**^**#**^
**%FEV1 predicted**^**a**^	**98.2 (25.7)**	**109.4 (21.1)**	**88.9 (25.4)**	**0.0001**^**#**^
**%IC**^**a**^	**85.9 (19.5)**	**83.6 (17.8)**	**87.9 (20.6)**	**0.111**
**chest CT finding**				
**emphysema**	**30.4 (82)**	**20.3 (25)**	**38.8 (57)**	**0.001**^**#**^

n indicates number.

^a^Data are shown as mean (SD).

All other data are shown as% (numbers).

^#^p < 0.05.

BMI: body mass index; VC: vital capacity; FEV1: forced expiratory volume in 1 second; FVC: forced vital capacity; IC: inspiratory capacity; GOLD: the Global Initiative for Chronic Obstructive Lung Disease.

### Association of COPD prevalence with lung cancer characteristics in Japanese patients undergoing bronchoscopy

To evaluate the association of COPD with characteristics of lung cancer, the pathological findings, EGFR mutation status, clinical staging, and decision for thoracic surgery were compared between the COPD group and the non-COPD group (Table 3).

**Table 3:** Characteristics of lung cancer status among non-COPD and COPD patients

	**All cases (n = 270)**	**Non-COPD (n = 123)**	**COPD (n = 147)**	**p value**
**Pathology**				**0.0001**^**#**^
**Adenocarcinoma**	**53.7 (145)**	**69.9 (86)**	**40.1 (59)**^**##**^	
**Sq**	**27.0 (73)**	**17.9 (22)**	**34.7 (52)**^**##**^	
**NSCLC**	**10.4 (28)**	**4.9 (6)**	**15.0 (21)**^**##**^	
**SCLC**	**6.7 (18)**	**5.7 (7)**	**7.5 (11)**	
**Large**	**2.2 (6)**	**1.6 (2)**	**2.7 (4)**	
**Clinical stage**				**0.046**^**#**^
**1A**	**25.6 (69)**	**30.8 (38)**	**21.1 (31)**	
**1B**	**13.7 (37)**	**13.0 (16)**	**13.6 (20)**	
**2A**	**9.6 (26)**	**13.0 (16)**	**7.5 (11)**	
**2B**	**7.8 (21)**	**9.8 (12)**	**6.1 (9)**	
**3A**	**11.9 (32)**	**8.9 (11)**	**14.3 (21)**	
**3B**	**5.6 (15)**	**4.1 (5)**	**6.8 (10)**	
**4**	**17.0 (46)**	**16.3 (20)**	**17.7 (26)**	
**ND**	**8.9 (24)**	**4.1 (5)**	**12.9 (19)**^**##**^	
**Thoracic surgery**				**0.0001**^**#**^
**Yes**	**138 (51.1)**	**64.2 (79)**	**40.1 (59)**	
**EGFR mutation status**				**0.001**^**#**^
**Yes**	**14.8 (40)**	**21.1 (26)**	**9.5 (14)**^**##**^	
**No**	**55.2 (149)**	**59.3 (73)**	**51.7 (76)**	
**ND**	**30.0 (81)**	**19.5 (24)**	**38.8 (57)**^**##**^	

n indicates number.

All data are shown as% (numbers).

ND indicates “not determined”.

^#^p < 0.05.

^##^indicates a significant difference compared with the non-COPD group.

COPD: chronic obstructive pulmonary disease; Sq: squamous cell carcinoma; NSCLC: non-small cell lung carcinoma; SCLC: small cell lung carcinoma; Large: large cell carcinoma; EGFR: epidermal growth factor receptor.

Regarding pathologic findings, the incidence of adenocarcinoma was significantly lower in the COPD group than in the non-COPD group. In the present study, EGFR mutation was observed only in the patients with adenocarcinoma (40/145 cases; 27.6%). In contrast, the number of cases in which EGFR mutation status was not determined was significantly higher in the COPD group than in the non-COPD group. Although determination of the clinical stage should be essential in order to propose the therapeutic options for lung cancer, some cases in which clinical staging had not been completed were observed in the study population. The number of these cases was significantly higher in the COPD group than in the non-COPD group. In contrast, the proportion of patients with each classification in the clinical staging did not differ between the two groups besides the cases in which the clinical staging was not determined. Among the total study population, the number of thoracic surgeries performed was significantly lower in the COPD group than in the non-COPD group.

### Critical impact of the severity of airflow obstruction on the decision to propose thoracic surgery with curative intent

To explore whether or not the severity of airflow obstruction might affect the decision to propose thoracic surgery with curative intent, patients at stage 3B and 4 were excluded from the analysis because they were not eligible for thoracic surgery. In addition, patients for whom the clinical staging had not been completed were also excluded. As a result, we evaluated data from 185 patients with lung cancer at stage 1A to 3A who underwent spirometry and bronchoscopy. These patients were subdivided into those who underwent thoracic surgery (135 cases) and those who did not (50 cases). The characteristics and spirometric data for the patients with or without thoracic surgery are summarized in Tables 4 and 5. The characteristics of lung cancer among these groups are also shown in Table 6. Univariate analysis identified a significantly lower frequency of thoracic surgery among the patients of greater age and with more severe airway obstruction, and advanced clinical staging. Univariate analysis also identified a significantly higher frequency of thoracic surgery among patients with adenocarcinoma. Finally, all of the factors with a significant association in the univariate analysis were applied to the multivariate model to identify variables independently associated with the decision for thoracic surgery. Multivariate analysis identified more severe airway obstruction, advanced clinical stagings, and higher age, as independent factors affecting the decision on thoracic surgery (Table 7).

**Table 4:** Patient characteristics in the thoracic surgery and non-thoracic surgery groups

	**All cases (n = 185)**	**Non-surgery (n = 50)**	**Surgery (n = 135)**	**p value**
**Cases**	**100 (185)**	**27.0 (50)**	**73.0 (135)**	**0.0001**^**#**^
**Age, years**^**a**^	**70.3 (39–88)**	**74.6 (62–80)**	**68.7 (39–86)**	**0.0001**^**#**^
**Sex, male**	**72.4 (134)**	**76.0 (38)**	**71.1 (96)**	**0.581**
**History of smoking**	**78.9 (213)**	**82.0 (41)**	**77.0 (104)**	**0.549**
**COPD diagnosed**^**b**^	**49.7 (92)**	**66.0 (33)**	**43.7 (59)**	**0.012**^**#**^
**COPD managed**^**c**^	**9.2 (17)**	**20.0 (10)**	**5.2 (7)**	**0.004**^**#**^
**COPD-related systemic comorbidities**	**57.3 (106)**	**60.0 (30)**	**56.3 (76)**	**0.739**
**Diabetes**	**20.0 (37)**	**30.0 (15)**	**16.3 (22)**	**0.061**
**Ischemic disease**	**7.6 (14)**	**10.0 (5)**	**6.7 (9)**	**0.532**
**Hypertension**	**40.5 (75)**	**40.0 (20)**	**40.7 (55)**	**1.000**
**Hyperlipidemia**	**11.4 (21)**	**10.0 (5)**	**11.8 (16)**	**0.799**

n indicates number.

^a^Data are shown as mean (range).

^b^indicates the patients who were firstly diagnosed as COPD at bronchoscopy.

^c^indicates the patients who had been diagnosed as COPD before bronchoscopy.

All other data are shown as% (numbers).

^#^p < 0.05.

COPD: chronic obstructive pulmonary disease.

**Table 5:** Physical assessment variables among patients having thoracic surgery and non-thoracic surgery

	**All cases (n = 185)**	**Non-surgery (n = 50)**	**Surgery (n = 135)**	**p value**
**BMI (kg/m**^**2**^**)**^**a**^	**22.2 (3.0)**	**22.3 (2.4)**	**22.1 (3.2)**	**0.760**
**Spirometric variables**				
**%VC**^**a**^	**108.8 (20.6)**	**104.5 (20.4)**	**110.4 (19.6)**	**0.031**
**FEV1 (ml)**^**a**^	**2126 (612)**	**1810 (591)**	**2242 (580)**	**0.0001**^**#**^
**FEV1/FVC**^**a**^	**67.6 (13.0)**	**62.7 (17.0)**	**69.5 (10.7)**	**0.01**^**#**^
**%FEV1 predicted**^**a**^	**101.4 (25.5)**	**93.7 (28.7)**	**104.3 (23.7)**	**0.020**^**#**^
**%IC**^**a**^	**87.8 (18.9)**	**85.3 (19.7)**	**88.7 (18.6)**	**0.314**
**Severity of** **airway obstruction**				**0.002**^**#**^
**Non-COPD**	**50.2 (93)**	**34.0 (17)**	**56.3 (76)**^**##**^	
**GOLD grade 1**	**34.6 (64)**	**44.0 (22)**	**31.1 (42)**	
**GOLD grade 2**	**10.8 (20)**	**10.0 (5)**	**11.1 (15)**	
**GOLD grade 3**	**4.3 (8)**	**12.0 (6)**	**1.5 (2)**^**##**^	
**GOLD grade 4**	**0 (0)**	**0 (0)**	**0 (0)**	
**Chest CT finding**				
**Emphysema**	**30.8 (57)**	**36.0 (18)**	**28.9 (39)**	**0.374**

n indicates number.

^a^Data are shown as mean (SD).

All other data are shown as% (numbers).

^#^p < 0.05.

^##^indicates a significant difference compared with the non-surgery group.

BMI: body mass index; VC: vital capacity; FEV1: forced expiratory volume in 1 second; FVC: forced vital capacity; IC: inspiratory capacity; GOLD: the Global Initiative for Chronic Obstructive Lung Disease.

**Table 6:** Characteristics of lung cancer among patients having thoracic surgery and non-thoracic surgery

	**All cases (n = 185)**	**Non-surgery (n = 50)**	**Surgery (n = 135)**	**p value**
**Lung cancer histology**				**0.028**^**#**^
**Adenocarcinoma**	**58.4 (108)**	**44.0 (22)**	**63.7 (86)**^**##**^	
**Sq**	**30.8 (57)**	**38.0 (19)**	**28.1 (38)**	
**NSCLC**	**5.4 (10)**	**10.0 (5)**	**7.4 (10)**	
**SCLC**	**3.2 (6)**	**8.0 (4)**	**1.5 (2)**^**##**^	
**Large**	**2.2 (4)**	**0 (0)**	**3.0 (4)**	
**Clinical stage**				**0.0001**^**#**^
**1A**	**37.3 (69)**	**26.0 (13)**	**41.5 (56)**	
**1B**	**19.4 (36)**	**14.0 (7)**	**21.5 (29)**	
**2A**	**14.6 (27)**	**10.0 (5)**	**16.3(22)**	
**2B**	**11.4 (21)**	**8.0 (4)**	**12.6 (17)**	
**3A**	**17.3 (32)**	**42.0 (21)**	**8.1 (11)**^**##**^	
**EGFR status**				**0.0001**^**#**^
**Yes**	**16.2 (30)**	**8.0 (4)**	**19.3 (26)**	
**No**	**61.6 (114)**	**28.0 (14)**	**74.1 (100)**^**##**^	
**ND**	**22.2 (41)**	**64.0 (32)**	**6.7 (9)**^**##**^	

n indicates number.

^a^Data are shown as mean (range). ^b^Data are shown as mean (SD).

All other data are shown as% (numbers).

ND indicates “not determined”.

^#^p < 0.05.

^##^indicates a significant difference compared with the non-surgery group.

Sq: squamous cell carcinoma; NSCLC: non-small cell lung carcinoma; SCLC: small cell lung carcinoma; Large: large cell carcinoma; EGFR: epidermal growth factor receptor.

**Table 7:** Multivariate analysis of independent factors in decision-making process for proposing thoracic surgery with curative intent

**Variables**	**Odds ratio**	**95% CI**	**p value**
**Severity of airflow obstruction**			**0.002**^**#**^
**Non-COPD versus GOLD grade 1**	**0.920**	**0.370–2.289**	**0.858**
**Non-COPD versus GOLD grade 2**	**1.085**	**0.268–4.386**	**0.909**
**Non-COPD versus GOLD grade 3**	**0.025**	**0.004–0.167**	**<0.0001**^**#**^
**Clinical staging**			**<0.0001**^**#**^
**Stage 1A versus stage 1B**	**1.084**	**0.326–3.602**	**0.895**
**Stage 1A versus stage 2A**	**0.679**	**0.185–2.491**	**0.559**
**Stage 1A versus stage 2B**	**0.705**	**0.172–2.895**	**0.628**
**Stage 1A versus stage 3A**	**0.062**	**0.019–0.202**	**<0.0001**^**#**^
**Age (per one year)**	**0.858**	**0.802–0.917**	**<0.0001**^**#**^

^#^p < 0.05.

## Discussion

This is the first study to evaluate the clinical impact of the prevalence and severity of COPD on a large cohort of Japanese patients with lung cancer who underwent bronchoscopy. Mounting evidence suggests that there is a close association between COPD and lung cancer [9,10,13]. For example, a case-control study by Young, et al. demonstrated a high prevalence of COPD in patients with newly diagnosed lung cancer [10]; however, their study population comprised only Caucasian ancestry, and nonsmokers with lung cancer were also excluded [10]. Although many lines of evidence suggest that EGFR mutations are more common among women, never-smokers, patients with adenocarcinoma-type lung cancer, and patients of East Asian ethnicity including Japanese [11,19], the association of COPD prevalence with EGFR mutations has not been fully evaluated. Indeed, 21.1% of our Japanese study population was non-smokers, 42.1% of whom had adenocarcinoma with EGFR mutation. Compatible with the distribution of pathological findings, there was significantly higher rate of EGFR mutation in the non-COPD group than in the COPD group (Table 3). Although a recent study by Loganathan, et al. also showed that 67% of 436 patients with newly diagnosed lung cancer had undergone spirometry prior to receiving treatment [9], our study analyzed the prevalence of COPD and its severity in 84.4% of patients with newly diagnosed lung cancer, mainly by performing spirometry at bronchoscopy. Furthermore, almost 50% of Loganathan, et al.’s population were women [9], whereas only 26.7% of our population were women. Epidemiologic surveys of cancers in Japan and the United States of America might support the different proportion of women patients with lung cancer between our study and their studies [20,21]. Although we previously demonstrated that 43.2% of the patients undergoing major lung resection had COPD (178/412 cases) [14], here the prevalence of COPD was found to be 54.4% in Japanese patients with lung cancer who underwent bronchoscopy. In the present population, 61.3% of men had COPD (122/199 cases), whereas only 35.2% of women had COPD (25/71 cases). In addition, 95.5% of men had a history of smoking in our population, whereas 67.6% of women were non-smokers. In contrast, the percentage of non-smokers among women with lung cancer was only 10.5% in the study of Loganathan et al. Thus, the lower prevalence of COPD in women with lung cancer might be explained by the high rate of non-smokers among women in our Japanese population [13,22]. Although de Torres, et al. demonstrated, by using a well-characterized cohort of patients with COPD, that the incidence of dense lung cancers decreased as the severity of the airflow obstruction at baseline increased [23], the severity of COPD in Japanese patients with newly diagnosed lung cancer was classified mainly as GOLD grade 1 and 2 rather than as GOLD grade 3. Furthermore, our data showed that most patients were newly classified with COPD (84.4%; 124/147 cases), compatible with the incidence of the severity of COPD shown above or previously [13,23]. It should be noted that in comparing patients undergoing thoracic surgery, COPD patients had an average postoperative stay that was 61% higher, and a 100% greater need of prolonged oxygen therapy, than patients without COPD, indicating the clinical impact of the coexistence of COPD [14].

The prevalence of COPD might increase in Japanese patients with lung cancer, whereas the impact of COPD-related systemic comorbidities is also increasingly recognized in clinical aspects of COPD [7]. Thus, whether or not the decision-making process involved in proposing the therapeutic management of lung cancer might be independently affected by COPD in patients with lung cancer remains elusive. To address this issue, we evaluated whether or not completion of clinical staging and proposal of thoracic surgery with curative intent might be affected by the coexistence of COPD. The percentage of patients in whom clinical staging had been not completed was significantly higher in the COPD group than in the non-COPD group. More than half of these patients were referred to other hospitals for further support, while the others were patients with disease recurrence. The proportion of patients with each classification in the clinical staging was compatible with that reported in previous studies about thoracic surgery [24]. Clinical guidelines recommend the assessment of spirometry to evaluate the optimum selection of surgical procedure in view of the risks of mortality and postoperative complications [6,8,25]. Therefore, we analyzed data from 185 patients with lung cancer at stage 1A to 3A because these patients are generally eligible for thoracic surgery with curative intent [17,26]. Even among these surgical candidates, however, the number of surgeries performed was significantly lower in the COPD group (64.1%; 59/92 cases) than in the non-COPD group (81.7%; 76/93 cases) (Table 4). Furthermore, our data showing that COPD-related systemic comorbidities might not be independent factors for proposing thoracic surgery with curative intent was supported by previous data as described above [14]. Thus, these data indicate that the decision-making process for the therapeutic management of Japanese lung cancer patients might be affected by the prevalence and severity of COPD. Finally, we evaluated whether or not the severity of COPD, classified by GOLD grade, might be an independent factor affecting the proposal of thoracic surgery with curative intent. Multivariate analysis indicated that severity of COPD was a critical and independent factor for proposing thoracic surgery with curative intent to Japanese patients with lung cancer who underwent bronchoscopy. This finding might be supported by our previous study showing that in comparing patients undergoing thoracic surgery, COPD patients with an FEV1/FVC below 0.70 had an average postoperative stay that was 61% higher, and a 100% greater need of prolonged oxygen therapy (POT), than patients without COPD [14].

Some limitations of the present study deserve mention. First, the reversibility test was performed in only 62.2% of patients (168/270 cases), although COPD was defined as a postpronchodilator FEV1/FVC below 0.7 [16]. This limitation is present in other studies that have evaluated the prevalence of COPD [9,10,27]. The other explanation might be the preoperative pulmonary assessment based on the clinical guidelines, in which the need to perform a reversibility test for assessment of airflow obstruction is not mentioned [8,25]. Although a recent study suggests that some COPD patients show relatively high reversibility for a short-acting beta2-agonist [28], only 1.2% of 168 cases showed significant reversibility in the present study, indicating that Japanese patients with both lung cancer and COPD might have different characteristics from that population [27]. Second, the present study retrospectively analyzed 270 out of a total of 320 cases with lung cancer in a single institution and therefore might be subject to selection bias. However, analyzing the data from 84.4% of all patients in a single institution who were sequentially registered and underwent bronchoscopy from 2010 to 2012 might minimize the possible contribution of the selection bias for patients with lung cancer.

Although many studies suggest that COPD remains underdiagnosed in the patients with lung cancer [13,14], Zang, et al. suggest that awareness of COPD might contribute the conformity to GOLD treatment guideline for stable condition and acute exacerbation of COPD in lung cancer patients during hospitalization [13]. When spirometry was performed at bronchoscopy, the median time from the date of spirometry to thoracic surgery was 50 days in the present study. Therefore, comprehensive assessment of COPD at bronchoscopy might allow us to implement the optimum management for lung cancer patients [29,30].

## Conclusions

In the present study, the high prevalence of COPD among Japanese patients with newly diagnosed lung cancer was shown. Although further investigation into the validity of the assessment of COPD at bronchoscopy from studies of patients with lung cancer from other institutions is warranted, we conclude that appropriate risk stratification and comprehensive management of patients with lung cancer and COPD might be made by assessment of the coexistence and severity of COPD at the time of bronchoscopy.

## Competing interests

The authors have declared that no conflict of interest exists.

## Authors’ contributions

NH, AM, and YH had full access to all of the data in the study and are responsible for the integrity of the data and the accuracy of the data analysis. YO: contributed to collection of the data. NI: contributed to interpretation of the study data. SI: contributed to interpretation of the radiological data. KW: contributed to the development of the analytic concept, data analyses. KI: contributed to interpretation of the study data. KY: contributed to critical revision of the manuscript. All authors read and approved the final manuscript.

## Pre-publication history

The pre-publication history for this paper can be accessed here:

http://www.biomedcentral.com/1471-2466/14/14/prepub
